# Hippocampal Signatures of Episodic Memory: Evidence from Single-Unit Recording Studies

**DOI:** 10.3389/fnbeh.2013.00054

**Published:** 2013-05-23

**Authors:** Amy L. Griffin, Henry L. Hallock

**Affiliations:** Department of Psychology, University of Delaware, Newark, DE, USA

**Keywords:** place cell remapping, trajectory coding, hippocampal-prefrontal synchrony, electrophysiology, dorsal hippocampus

## Abstract

What hippocampal neural firing patterns signal memory and, more importantly, how is this memory code used by associated structures to translate a memory into a decision or action? Candidate hippocampal activity patterns will be discussed including (1) trajectory-specific firing of place cells with place fields on an overlapping segment of two (or more) distinct trajectories (2) prospective firing of hippocampal neurons that signal an upcoming event or action, and (3) place cell remapping to changes in environment and task. To date, there has not been compelling evidence for any of these activity patterns being the neural substrate of episodic memory. New findings suggest that learning and memory processes are emergent properties of interregional interactions and not localized within any one discrete brain region. Therefore, the next step in understanding how remapping and trajectory coding participate in memory coding may be to investigate how these activity patterns relate to activity in anatomically connected structures such as the prefrontal cortex.

## Introduction

For decades scientists have been attempting to understand how memories are made, stored, and retrieved in the brain. Unraveling this problem is not only fascinating in its own right, but can lead to the development of treatments for a multitude of disorders and conditions that affect the ability to form and use memories in our day-to-day experiences. In recent years, the development of large-scale neural recording techniques has advanced our knowledge of neural underpinnings behind memory formation and retrieval. However, a challenge that remains in the field is the large degree of uncertainty in linking neural firing patterns with complex cognitive operations. For that reason, despite the many investigations of the neural correlates of memory, much ambiguity remains regarding which brain structures are involved and the nature of their involvement. An extensive body of literature has established the hippocampus as a critical brain structure in episodic memory. Hippocampal lesions (or disruption of hippocampal inputs) lead to performance impairments in tasks that rely on the encoding and retrieval of specific trajectories through a familiar environment. These tasks include the delayed spatial alternation task (Rawlins and Olton, 1982; Brito et al., 1983; Stanton et al., 1984; Aggleton et al., 1995; Czerniawski et al., 2009), the eight-arm radial maze (Olton et al., 1979), and the Morris water maze (Eichenbaum et al., 1990). Recording studies, in turn, are beginning to uncover the exact mechanisms utilized by the hippocampus to accomplish memory processing. The most direct way to study cellular mechanisms that support episodic memory is to record from populations of neurons while animals perform memory tasks. Candidate neural activity patterns that have been linked to episodic memory will be discussed in this review including (1) trajectory-specific firing of place cells with place fields on an overlapping segment of two (or more) distinct trajectories (2) prospective firing of hippocampal neurons (3) place cell remapping to changes in environment and task. Though these phenomena may indeed serve as a neural substrate for episodic memory, a complex process like episodic memory most likely relies on functional interactions among a network of brain regions. Therefore, developing an understanding of these hippocampal activity patterns in the broader context of network interactions could be a critical step in identifying the neural correlates of memory.

## Hippocampal Involvement in Working Memory and Spatial Cognition

Spatial working memory tasks have been an essential tool for developing rodent models of memory. However, during these tasks, there are presumably several processes at work: working memory, the temporary storage of information that is necessary for complex cognitive processes (Baddeley, 1992); spatial cognition, the development and use of a “cognitive map” of the environment (O’Keefe and Nadel, 1978) and episodic encoding and retrieval of specific trajectories through the environment (Hasselmo, 2009). In a typical working memory task, the spatial alternation task, rats are placed on an elevated T-maze and required to alternate between the left and right goal arms on each trial. The task relies on the rat’s ability to remember which goal arm was visited on the previous trial in order to correctly select the opposite goal arm. There are two main versions of this task: continuous alternation, in which the rat alternatively visits the left and right goal arms in a “figure 8” pattern, and delayed alternation, in which the rat also alternates visits to the left and right goal arm, but pauses in the start box between trials. Because the insertion of the delay period necessitates remembrance of the previously rewarded goal location, the memory demand is theoretically greater for delayed version of the alternation task than for the continuous version. Accordingly, hippocampal lesions (or disruption of its inputs) lead to performance impairments in delayed alternation (Rawlins and Olton, 1982; Brito et al., 1983; Stanton et al., 1984; Ainge et al., 2007a; Czerniawski et al., 2009), but not continuous alternation (Ainge et al., 2007a). Although these studies have elucidated the brain regions that are necessary for memory, it is unclear if the results can be extrapolated to episodic memory in humans. In fact, episodic memory has been explicitly defined as having a “what” component, a “when” component, and a “where” component and as a process that is not present in animals other than humans (Tulving and Markowitsch, 1998). However, there is strong evidence that episodic memory is not a uniquely human phenomenon. Clayton and Dickinson (1999) have demonstrated that Western scrub jays can remember not only where a food cache was stored, but what type of food it was and how long ago it was cached. The type of memory that includes spatial, temporal information is often called “episodic-like” memory when applied to experimental animals. Fortin et al. (2002) demonstrated that “episodic-like” memory depends on the hippocampus by showing that rats with hippocampal lesions were unable to perform a sequential odor task. Rats were presented with a sequence of five odors and after a 3-min delay were presented with two of the odors and were required to identify which was presented earlier in the sequence. Rats with hippocampal lesions were impaired on this task, but importantly were not impaired on a probe recognition task in which they were required to discriminate between novel and familiar odors. Although this study demonstrated that rats could form hippocampus-dependent “what–when” representations, the “where” component was missing. Subsequent studies showed that rats (Ergorul and Eichenbaum, 2004) and mice (DeVito and Eichenbaum, 2010) were significantly impaired on tasks that required the integration of “what,” “when,” and “where” information following hippocampal damage. Together, these lesion studies have established the hippocampus as a critical brain region in many types of memory, including episodic memory. In order to examine the physiological properties that give rise to episodic memory, we must turn to studies that have recorded populations of hippocampal neurons in freely moving rats.

## Spatial Coding in Hippocampal Neurons

Hippocampal neurons known as place cells code spatial location by showing selective elevations in firing rate when the rat occupies specific locations in an environment (O’Keefe and Nadel, 1978). Although the discovery of hippocampal place cells was a significant advancement in the understanding of hippocampal physiology, it has been difficult to reconcile the human clinical findings that the hippocampus was critical for (non-spatial) episodic memory relative to the rodent findings, which suggested that the hippocampus was a “cognitive map” of the environment and thus participated solely in spatial processing. Additional research soon revealed that current spatial location was not the only factor that modified the behavior of place cells. McNaughton et al. (1983) found that the firing rate of a given place cell could be influenced by the direction in which a rat was heading when the rat passed through the neuron’s place field. This place cell “directionality” was observed when rats moved through a radial arm maze, such that a given place cell would show a significantly different firing rate when the rat was headed toward the end of an arm (outbound journeys) than when the rat was headed toward the center of the maze (inbound journeys), and vice versa. This within-field directional coding provided early evidence that principal cells in the hippocampus could respond to an animal’s previous and upcoming location in addition to its current location in an environment. However, place cell directionality only appeared under certain experimental conditions. When rats foraged for food in a circular or square open-field enclosure, the firing rates of place cells did not differ significantly as a function of the future or past position of the animal (Breese et al., 1989; Muller et al., 1994). Place cell directionality was again seen when rats performed a spatial navigation task in a radial arm maze, but not when rats performed a non-spatial odor discrimination task (Wiener et al., 1989). These results suggested that directional coding in the hippocampus only appears when an animal moves through a place field in a stereotyped manner, such as when an animal’s trajectory through a place field is limited by the experimental apparatus in which testing is taking place.

Could the physical boundaries of an environment be the sole factor in determining whether place cell firing could be modified by an animal’s direction? Markus et al. (1995) first recorded place cells while rats navigated through a radial arm maze, and then recorded place cells as rats foraged in an open cylinder. Predictably, the experimenters found that directional coding of place cells was observed in the radial arm maze, but not the open cylinder. However, when the task contingency in the open cylinder was altered so that the animals no longer foraged for randomly distributed food rewards, but were taught to run to the periphery of the cylinder toward reward zones that were sequentially baited, place cells began to display the same directionally modified properties that were observed in the radial arm maze. Thus, directional coding could be influenced by task strategy, even when the task was performed in an open-field environment.

## Place Cell Remapping

Place cells are known to exhibit radical changes in firing properties with sometimes subtle changes in the features of an environment, a property known as “remapping” (for review Muller et al., 1996; Colgin et al., 2008). Operationally, remapping is defined as a change in firing rate and/or place field location in the “new” environment. These changes can manifest themselves in a number of ways: A place cell that ceases to fire; a previously silent place cell that begins to fire; or a place field that shifts to an entirely new location within the environment. The first demonstration of remapping was shown in a study by Muller and Kubie (1987). It was found that doubling the area of a circular or square enclosure caused a subset of place cells to remap. Similarly, in this same study, a population of hippocampal neurons was recorded while rats foraged in both circular and square environments. There was no relationship between the firing field locations in one enclosure and the field location in the other enclosure, suggesting that the hippocampal ensemble had a separate representation for each environment. Following this initial demonstration, Bostock et al. (1991) used a black or white cue card as a polarizing cue in an otherwise-identical recording chamber. The first time that the black card was replaced by the white card, few place cells changed. But, after alternating between the two cards several times, the place cells began to discriminate between the two “environments,” with some cells shifting their place field location, some ceasing to fire in one environment and some showing “complex” or “global” remapping: changing in both location and shape. It was hypothesized that remapping allowed the rat to disambiguate similar environments; reducing interference by having populations of hippocampal neurons alter their firing correlates between conditions. This demonstration of remapping after experience with an environment was also seen in investigations of remapping between circular and square enclosures. Lever et al. (2002) recorded the same neurons in two separate enclosures that differed only in shape of the exterior walls: circular or square. As in the Bostock study, the first exposure to the changed environment did not induce a change in the place field locations or shape. However, the neurons began to discriminate between environments after multiple switches. Wills et al. (2005) morphed the recording enclosure gradually from a square to a circle in order to investigate further how hippocampal neurons differentiate between circular and square enclosures. Most hippocampal neurons showed remapping and the remapping was abrupt and consistent across simultaneously recorded cells, suggesting that the hippocampal network codes changes in the environment in a coherent manner. Importantly, it is not only sensory changes in the environment that can induce remapping. As described above, Markus et al. (1995) found that requiring the rat to switch from a foraging strategy to a goal-directed strategy in the same enclosure induced remapping. Similarly, Moita et al. (2004) showed remapping based on whether fear conditioning was performed in the environment.

**Figure 1:**
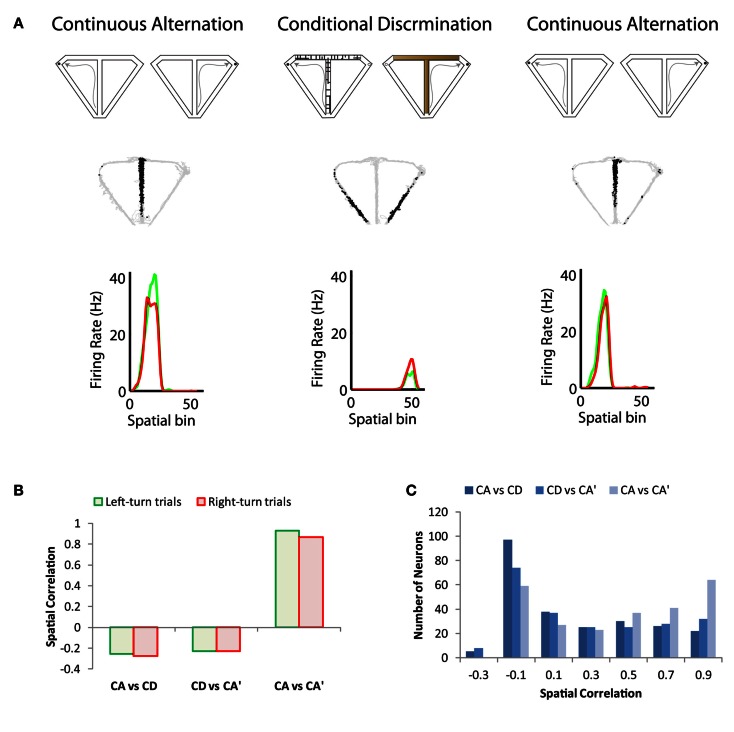
**Illustration of task remapping exhibited by dorsal CA1 hippocampal neurons**. **(A)** The top panel is a schematic of the continuous alternation and conditional discrimination tasks. Rats were trained on both tasks prior to implantation of recording microdrives. Recoding sessions consisted of a set of continuous alternation trials, followed by a set of conditional discrimination trials, followed by a second set of continuous alternation trials. The middle panel shows the trajectory of the rat (gray) with superimposed spike locations (black) during the first set of continuous alternation trials (CA, left), a set of conditional discrimination trials (CD, middle), and second set of continuous alternation trials (CA’, right). The neuron has a prominent place field on the central stem of the T-maze during both sets of continuous alternation trials, which remaps to the return arms of the T-maze during the set of conditional discrimination trials. The bottom panel shows the average firing rate for left (green) and right (red) trials across 5-cm spatial bins of the T-maze. Spatial bins 1–24 correspond to the central stem region of the maze; 25–26 to the choice point; 27–36 to the goal arm; and 37–55 to the return arm. The neuron does not exhibit trajectory coding as shown by the similar firing rate distributions on left- and right-turn trials. **(B)** Spatial correlation of bin firing rates across the continuous alternation and conditional discrimination tasks for the neuron shown in **(A)**. The spatial correlation is low between the conditional discrimination task and both sets of continuous alteration trials (CA vs. CD and CD vs. CA’), which indicates strong remapping. Conversely, the spatial correlation is high between the two sets of continuous alternation trials (CA vs. CA’). **(C)** Spatial correlation values across tasks for a population of recorded dorsal CA1 neurons. The population showed the same task remapping pattern as the neuron in **(A)**: high correlation values between sets of continuous alternation trials and low correlation values across the conditional discrimination and continuous alternation tasks. Data adapted from Hallock and Griffin (2013).

Recent studies have shown place cell remapping in response to changes in task (in the absence of changes in the spatial layout of the environment). Ferbinteanu et al. (2011) showed that switching from a cue-guided strategy to a spatial strategy prompted place cell remapping. Importantly, the overt behavior of the rat was the same in the two different tasks; only the memory demand differed. Ainge et al. (2012) compared prospective coding across behaviorally identical tasks and showed no differences in the coding behavior of hippocampal neurons between the memory-guided and cue-guided conditions. Conversely, a recent study showed that a large percentage of hippocampal neurons remapped between continuous alternation and conditional discrimination tasks, a phenomenon that was termed “task remapping” (See Figure 1; Hallock and Griffin, 2013). When a delay was added to the alternation task, however, there was very little remapping between tasks, suggesting that the temporal structure of the task (discrete vs. continuous trials) was driving the place cell remapping rather than the memory demand of the task (Hallock and Griffin, 2013).

At this point, it is important to distinguish between the two types of remapping seen in hippocampal neural populations. Rate remapping, defined as a significant difference in place field firing rate without a change in place field location, was first reported in response to “local” changes in an environment: changing the wall color or shape of the recording enclosure within the same room (Leutgeb et al., 2005). In contrast, global remapping, defined as changes in place field locations and firing rate, is induced by either physically moving the rat between recording rooms as in Leutgeb et al. (2005) or by changing the recording environment substantially (e.g., Wills et al., 2005). Rate remapping theoretically allows the hippocampus to code different experiences that occur in the same location, whereas global remapping allows similar experiences to be distinguished based on where the experience took place (Leutgeb et al., 2005). The findings showing that place cells do not exclusively code spatial location have led many to speculate that remapping could be a mechanism for linking a spatial location (the “where”) to events (the “what” and the “when”) occurring in that location (Colgin et al., 2008). Formation of this “what-when-where” representation is a critical component of episodic memory.

## Trajectory Coding in Hippocampal Neurons

Evidence for directional coding and remapping in the hippocampus added another chapter to the cognitive mapping theory of hippocampal function. Place cells could be tied to the current, past, or future spatial location. Directionally dependent firing in the hippocampus also added a possible clue of how hippocampal neurons participated in the formation, storage, and retrieval of contextually unique events that comprise episodic memories. Similar to the argument that place cell remapping could be a mechanism for linking the “where” with the “what” and the “when,” the reasoning was that if place fields are present in one situation (e.g., the rat is moving in a particular direction) and absent in another situation, the neurons cannot exclusively be coding spatial location (Wood et al., 2000). It was tempting to speculate that the neurons could be coding memory on top of a place code. Since the pioneering studies on the amnesic patient H.M., who had undergone a surgical procedure to remove large portions of his medial temporal lobe in order to control seizure activity, it had been known that the hippocampus was critical for the formation of new episodic memories (Scoville and Milner, 1957). However, evidence from single-unit recording of hippocampal neurons in animals suggested that the primary role of the hippocampus was spatial processing (O’Keefe and Nadel, 1978). Although spatial mapping and episodic memory are not mutually exclusive, there are many aspects of memory that are non-spatial. Using a continuous non-matching to sample odor task, Wood et al. (1999) found that the majority of recorded hippocampal neurons (∼85%) coded for non-spatial variables such as odor, trial type (match vs. non-match), approaching the stimulus cup, or a conjunctive coding of these non-spatial variables with location. These findings suggested that the hippocampus represents both spatial and non-spatial information related to memory. Subsequent studies by Frank et al. (2000) and Wood et al. (2000) began to tease apart the cognitive mapping and episodic memory functions of the hippocampus by recording from hippocampal neurons during tasks in which an animal was required to remember a previously visited location in order to successfully retrieve a reward on an upcoming trial. Wood et al. (2000) showed that when rats ran a continuous spatial alternation task in a T-maze, the majority of neurons with place fields on the stem of the maze showed a significantly higher firing rate during either left- or right-turn trials. Frank et al. (2000) added to this line of research by showing that place cell firing rate could be tied to both past and future location by recording in a W-maze as rats alternated between the two outside arms via the central arm. This study revealed that firing rate was modulated both during inbound journeys through the central arm (indicative of retrospective coding), and outbound journeys (indicative of prospective coding).

Providing further evidence that hippocampal neurons could flexibly code for both future and past position, Ferbinteanu and Shapiro (2003) recorded from dorsal hippocampus while rats performed a spatial memory task in a plus maze, in which starting location and goal location could be varied. Neuronal firing rate was heavily modified by the trajectory of an animal, with neurons that fired on a start arm selectively signaling journeys to a specific goal arm, and neurons that fired on a goal arm selectively signaling journeys from a specific start arm. Hippocampal neurons display similar trajectory-dependent coding in a maze that has multiple choice points within a journey, as shown during recordings on a concatenated Y-maze (Ainge et al., 2007b). When a delay period is introduced between trials of the T-maze continuous alternation task, neurons cease to show trajectory-specific coding on the maze stem. Instead, neurons that fire during the delay period show firing rates that are modulated by the past or future location of the animal, indicating that trial-specific activity takes place at the location where memory coding that is necessary for contextual disambiguation is most likely to happen (Ainge et al., 2007a; Pastalkova et al., 2008). It has been theorized that this type of trajectory coding is a neural mechanism for memory processing, as hippocampal neurons can fire at different rates in a common location as a function of the animal’s future or past position, and thus separate distinct events that occur in common spatial locations (Hasselmo and Eichenbaum, 2005; Smith and Mizumori, 2006; Griffin et al., 2007).

However, evidence from other experiments has challenged the idea that trajectory coding is important for distinguishing between events that contain common features. In one study, trajectory coding was not found as rats alternated continuously between arms of a Y-maze (Lenck-Santini et al., 2001). When rats are running on a circular track, trajectory coding is seen in larger proportion when local cues are present, indicating that levels of sensory input can influence bidirectional coding (Battaglia et al., 2004). Bower et al. (2005) showed that trajectory coding was not necessary for the successful performance of a task in which rats had to disambiguate between journeys that contained repeating elements that were common between different trajectories. Interestingly, trajectory coding did appear in this study, but only under certain circumstances; specifically, when removable barriers were introduced during training, and when rewards were withheld at intermediate steps and only given at the end of a trajectory. Further challenging the notion that trajectory coding is a hippocampal-dependent memory signal, trajectory coding appears in tasks that are not dependent on the integrity of the hippocampus (Wood et al., 2000; Lee et al., 2006; Ferbinteanu et al., 2011; Griffin et al., 2012). Trajectory coding is seen in equal proportion during a spatial task on a plus maze that is hippocampus-dependent, and a cue-approach task on a plus maze that is not hippocampus-dependent (Ferbinteanu et al., 2011). Trajectory coding is seen in a large proportion of hippocampal neurons during the continuous alternation task, which is not dependent on the functional integrity of the hippocampus (Wood et al., 2000; Lee et al., 2006; Griffin et al., 2012). Other studies have found that trajectory coding is not seen during a cue-approach task in a radial arm maze (Berke et al., 2009) and conditional discrimination tasks in T-mazes (Ainge et al., 2012; Griffin et al., 2012). These mixed results indicate that trajectory coding is not simply a mechanism for context-specific encoding in episodic memory, but is rather a complex phenomenon that is influenced by a variety of sensory and behavioral components of experiences.

More recent studies have begun to unravel the links between experience and trajectory coding by manipulating task, duration of exposure, and visual cues surrounding the recording environment. When rats switch from a well-known task strategy to a novel strategy in a W-maze, retrospective coding is seen before rats reach performance levels above chance on the novel task (Ji and Wilson, 2008). In agreement with this finding, Bahar and Shapiro (2012) found that when the goal arm was switched during a well-learned spatial task in a plus maze, prospective and retrospective trajectory coding stayed consistent even when animals were not yet able to perform the new variation of the task. In contrast, when the arrangement of visual cues in the recording room was significantly altered, trajectory coding disappeared and only returned when rats had oriented to the new environmental layout. Upon introduction to a circular track, hippocampal neurons show little directionally dependent variation in firing rate; as rats gain more experience on the track, trajectory coding develops over time (Navratilova et al., 2012). Finally, when rats are trained on both continuous alternation and conditional discrimination in a T-maze, trajectory coding on the maze stem is virtually absent when the rat switches between the two tasks, indicating that experience during task training has a large influence on whether or not trajectory coding will be observed (Hallock and Griffin, 2013). In this same study, prospective trajectory coding was seen when rats switched between delayed alternation and conditional discrimination tasks on the same T-maze. This prospective coding was only observed during the delay period of the delayed alternation task, suggesting that when a delay period is introduced that increases the hippocampal-dependent memory demand of a task, hippocampal neurons may be more likely to display trajectory coding, even when previous training experience would not otherwise favor its development.

The initial discovery that the firing rate of place cells was influenced by the rats’ specific trajectory and thus by recent experiences led to speculation that this firing rate difference could be a “rate code” for episodic memory. However, this notion was challenged by the finding that trajectory coding was rarely observed in hippocampus-dependent tasks. After over a decade of research, the debate over whether trajectory coding represents a hippocampal memory signal or perhaps a broader phenomenon that encompasses training history and the structure of the task has not been resolved. More experiments will need to be completed in order to delineate the determining factors that produce trajectory coding during different tasks in different environments. It is clear, however, that both trajectory coding and remapping reflect coding mechanisms for distinguishing between tasks and environments. Remapping tends to occur with changes to the recording environment or task and trajectory coding is a specific type of remapping that occurs within a task when there are continuously overlapping paths toward different goal locations. In order to gain a true appreciation for the content of the information processed by the hippocampus, it may be fruitful to look outside of the hippocampus proper and explore the manner in which the contextual information is communicated to anatomically connected structures.

## Hippocampal–Prefrontal Interactions during Memory Processing

Emerging evidence suggests that the neural signature of complex cognitive functions may not reside within an individual brain structure, but in the dynamic interactions that take place within system of related structures. Consistent with this notion, a functional imaging study in humans demonstrated selective activation of both the hippocampus and PFC during a memory task (Stern et al., 2001). The interactions between hippocampus and prefrontal cortex are of particular interest because (1) these structures have been shown to be coactive during memory tasks; (2) there are direct and indirect anatomical connections between them and (3) there is emerging evidence for hippocampal–prefrontal interactions during simple cognitive tasks. Disruption of hippocampus–mPFC interactions may result in failed transfer of spatial and contextual information processed by the hippocampus to the circuitry in mPFC responsible for decision making and goal-directed behavior (Colgin, 2011; Gordon, 2011). The mPFC is known to receive direct monosynaptic glutamatergic input from CA1 and subiculum of hippocampal formation (Ferino et al., 1987; Jay and Witter, 1991; Jay et al., 1992). From this pattern of connections, it is tempting to conclude that the functional interactions between the hippocampus and prefrontal cortex are an important component of complex behavior. Despite many demonstrations of individual contributions of hippocampus and mPFC to memory-guided behavior, the interactions between these two brain regions during complex tasks has not yet been well studied. One way to directly observe a functional interaction is to measure oscillatory synchrony, changes in activity patterns within a specific frequency range that occur simultaneously in disparate brain regions. Two measures of synchrony are *coherence*, a measure that reflects the strength of the temporal relationship between two oscillations and *entrainment*, a measure of the consistency with which action potentials from a neuron in one region occur on a particular phase of an oscillation in another region. In general, theta synchrony appears to be a mechanism used by the hippocampus to convey information to anatomically connected structures, including the mPFC (Siapas and Wilson, 1998; Hyman et al., 2005; Jones and Wilson, 2005; Siapas et al., 2005; Benchenane et al., 2010), as well as the amygdala (Seidenbecher et al., 2003; Popa et al., 2010) and the striatum (Berke et al., 2004; Tort et al., 2008). Hippocampal theta is one of the few sustained oscillations in the brain. It is a ∼8 Hz oscillation dominating the hippocampal local field potential during exploration in the rodent (Buzsáki, 2002). Slow oscillations like the hippocampal theta rhythm are well-suited to coordinate interactions between disparate brain regions because the length of the theta cycle is sufficient to accommodate long conduction delays and even polysynaptic interactions. mPFC neurons exhibit strong entrainment to the hippocampal theta rhythm during exploration (Siapas and Wilson, 1998; Hyman et al., 2005) and spatial working memory (Jones and Wilson, 2005; Hyman et al., 2010;), suggesting a functional interaction between hippocampus and mPFC that may be especially important in situations in which demands on working memory are high. A recent study examined hippocampal–prefrontal interactions by performing lesions of the hippocampus and recording from mPFC neurons during a conditioned place preference task in which rats were required to wait in a goal zone before receiving food reward (Burton et al., 2009). Single mPFC neurons showed anticipatory activity during the wait time in the goal zone. Importantly, this activity was diminished in hippocampal-lesioned rats and this disruption was accompanied by impairments in task performance. This anticipatory activity may represent the expectation of forthcoming events. The disruption of this activity in hippocampal-lesioned rats suggests that hippocampal input may provide the mPFC with contextual information that is necessary for the selection of appropriate responses. A crucial next step in this line of research is to examine the link between trajectory coding, remapping, and hippocampal-prefrontal synchrony. It is reasonable to predict that hippocampal neurons that exhibit trajectory coding or remapping would be preferentially entrained to prefrontal activity when memory demand is high, confirming that the neural activity patterns that encode memories encompass a network of interconnected brain regions. Finally, the only way to address the issue of whether there is a causal link between neurophysiological phenomena and memory is to use multiple technical approaches. Therefore, a future direction in the area of memory research should be to combine techniques that measure neural activity patterns such as neurophysiology with inactivation techniques such as pharmacological inactivation of discrete brain regions (e.g., Brandon et al., 2011). The question of whether trajectory coding and remapping persist after disruption of the hippocampal-prefrontal circuit remains an open question.

## Conclusion

Hippocampal place cell remapping has been demonstrated most commonly in open-field environments. In these environments, the rats are not required to perform any specific task to obtain food reward, but instead must forage for food pellets scattered across the floor. The fact that place cell remapping can be driven not only by changes in the layout of an environment, but also by experiences within that environment suggests that remapping could play a key role in the formation of an episodic memory by linking the “what” and the “when” with the “where.” However, because studies that have reported global remapping have manipulated the sensory environment, the evidence so far suggests that remapping is the code for a context change; not an episode within that context. Rate remapping is more often associated with episodic memory in the literature. However, the evidence is simply insufficient at the stage to make statements about its role in episodic memory.

A number of investigations have recorded hippocampal neurons during memory task performance in apparatuses that restrict the rats’ movement to a path or trajectory. These tasks include spatial alternation (both delayed and continuous), serial reversals, delayed non-match to position/place, complex sequence tasks, and visuospatial conditional discrimination. Reminiscent of the changes seen in place cell firing properties in response to sensory or cognitive information, some, but not all, of these studies have found that hippocampal neurons code specific trajectories. The most robust demonstrations of trajectory coding have been seen in continuous spatial alternation (Wood et al., 2000; Lee et al., 2006) and serial reversal tasks (Ferbinteanu and Shapiro, 2003). Interestingly, some tasks that are known to depend on the hippocampus, such as delayed spatial alternation do not elicit robust trajectory coding (Ainge et al., 2007a; Griffin et al., 2012; Hallock and Griffin, 2013). This set of findings argues against the interpretation of trajectory coding being a memory signal used in task performance. Instead, trajectory coding may be a special case of remapping, in which the hippocampal network alternates between representing two or more different trajectories. The next step in understanding how remapping and trajectory coding participate in memory coding may be to look outside of the hippocampus in downstream structures such as the mPFC. By investigating mPFC–hippocampal interactions and synchrony during memory task performance and, most importantly, relating these interactions to trajectory coding and remapping of hippocampal neurons, we may finally uncover the meaning of these striking hippocampal firing patterns.

## Conflict of Interest Statement

The authors declare that the research was conducted in the absence of any commercial or financial relationships that could be construed as a potential conflict of interest.
